# Computer assisted skull base surgery: a contemporary review

**DOI:** 10.1515/iss-2021-0020

**Published:** 2022-06-30

**Authors:** Alexander K. Bartella, Steven G. Hoshal, Bernd Lethaus, E. Bradley Strong

**Affiliations:** Department of Oral and Maxillofacial Surgery, Leipzig University Leipzig, Germany; Department of Otolaryngology – Head and Neck Surgery, University of California, Davis, Sacramento, CA, USA

**Keywords:** 3D printing, augmented reality, computer aided surgery, intraoperative CT, navigation, virtual reality, VSP

## Abstract

Skull base surgery has evolved significantly since Harvey Cushing‘s first descriptions in the early 1900s. Computer aided surgery (CAS) applications continue to expand; they include virtual surgical planning, augmented and virtual reality, 3D printing of models/cutting guides/implants, surgical navigation, and intraoperative imaging. The authors will review the current skull base CAS literature and propose a computer aided surgical workflow categorizing these applications into 3 phases: 1) Virtual planning, 2) Surgical execution, 3) Intraoperative verification.

## Introduction

The first approaches to the anterior skull base were described by Cushing and Hirsch in the early 1900s [1, 2]. Smith and Maleki are attributed with some of the earliest open craniofacial approaches to the skull base in the 1950s [3, 4]. Because skull base surgery involves vital, complex anatomy in a limited physical space, surgical advances have often mirrored technological advances. The application of endoscopy and computed tomography (CT) in the 1970s improved surgical planning and accuracy [5], [6], [7], [8]. In the late 1990s the introduction of surgical planning software, 3D printing, and intraoperative navigation/CT expanded the scope of skull base surgery to include the entire anterior and middle cranial fossa [9], [10], [11], [12], [13], [14], [15], [16], [17], [18], [19], [20], [21], [22]. More recent refinements in intraoperative navigation and CT have resulted in decreased operative times and improved clinical outcomes [13, 14, 23]. Augmented and virtual reality tools have shown efficacy in resident education and presurgical planning [10, 24]. The authors review the current literature and present a working paradigm for computer aided skull base surgery.

## Methods

The authors conducted a Pubmed.gov literature review from 2011 to 2021 using the key words “Virtual Planning” AND “Skull Base Surgery”; “Computer Aided Planning” AND “Skull Base Surgery”, and “Skull Base Surgery”. Only case series with >3 patients were considered. Cohorts describing orthognathic surgery were excluded. A total of 16 articles were identified (Table 1) and are included in the review.

**Table 1:** Overview of literature addressing computer assisted skull base surgery from 2011 to 2021. Articles are ordered by their year of publication.

Author/Publication	Year/Journal	Kind of article	Anatomical location	Content	Preoperative planning	Execution	Verification	n patients
Novak et al./The use of an O-arm in endonasal endoscopic operations of the skull base (43)	2021 BMC Surgery	Prospective pilot study	Hypophyseal adenoma	Neuronavigation with additional intraoperative 3D X-ray examination had a significant lower error	MRI and CT scans	Intraoperative 3D X-ray examination	With intraoperative navigation: 0 mm deviation, without 2.65 mm	6
Swendseid et al./VSP in subscapular system free flap reconstruction of midface defects (39)	2020 Journal of Oral Oncology	Retrospective cohort study	Mid-face	Virtual planning in reconstructive mid-face surgery	Commercial planning CT scan	Commercial planning and cutting guides	Postoperative CT scan and calculation of the deviation.	Nine patients with surgical planning and 14 without
Mounir et al.: Computer-guided gap arthroplasty: a New approach to the execution of preplanned osteotomies for the treatment of bony ankylosis of the temporomandibular joint	2020 British Journal of Oral and Maxillofacial Surgery	Case series	TMJ surgery	Usability of a 3D printed cutting guide in TMJ arthroplasty	CT scan, MIMICS (medical 19.0 materialise),	3D printed cutting guide	CT scan and comparison with preoperatively defined fixed points	5
Merema et al./Accuracy of fit analysis of the patient-specific groningen temporomandibular joint prosthesis (44)	2020 International Journal of Maxillofacial Surgery	Research paper	TMJ surgery	Total joint replacement IPS	Prosthetsis in 10 patients CT scan	Surgical guides, Patient specific implant	Deviation of 1.07 mm from preoperative planning	11
Boccalatte et al.: Computer-assisted surgery for replacement of the temporomandibular joint with customized prostheses: can we validate the results? (45)	2020 Oral Maxillofacial Surgery	Case series	TMJ surgery	Joint replacement via IPS	Virtual planning CT, MRI, SPECT (condylar hyperplasia), wax up	Virtual navigation Surgical splints (3D printed)	Postoperative overlap of screws planned/executed Deviation 2.08 mm	6
Zheng et al.: Customized skull base-temporomandibular joint combined prosthesis with 3D-printing fabrication for craniomaxillofacial reconstruction: a preliminary study (46)	2019 International Journal of Maxillofacial Surgery	Prospective case series	TMJ surgery	Joint replacement via IPS	Virtual planning simulation of Resection CT	PSI, No cutting guide, no navigation	Improvement of clinical indices (pain, etc.)	5
Siegmund et al.: Reconstruction of the temporomandibular joint: a comparison between prefabricated and customized alloplastic prosthetic total joint systems (47)	2019 International Journal of Maxillofacial Surgery	Clinical paper	TMJ surgery	Stock vs. IPS TMJ replacement	IPS (Biomet) vs. Stock; DICOM data set to Biomet		Clinical improvement, less complications in IPS	28 (16 IPS, 12 stock)
Zheng et al.: An innovative total temporomandibular joint prosthesis with customized design and 3D printing additive fabrication: a Prospective clinical study (48)	2019 Journal of Translational Medicine	Prospective case series	TMJ surgery	IPS joint replacement	CT scan, 3D model, digital design of the components, fitting on the 3D printed skull	Surgical templates	Postop CT scan, clinical and subjective data	12
Dimitroulis et al.: A new three-dimensional, print-on-demand temporomandibular prosthetic total joint replacement system: Preliminary outcomes (49)	2018 Journal of Cranio-Maxillofacial Surger	Prospective cohort study	TMJ surgery	IPS total joint replacement	CT scan		Clinical evaluation	38 patients/50 devices
Bradley et al.: Intraoperative three-dimensional virtual reality and computed Tomographic guidance in temporomandibular joint arthroplasty of syndromic craniofacial dysostoses (35)	2019 Journal of Plastic and Reconstructive Surgery Global open	Case series	TMJ surgery	Virtual reality in planning of syndromatic patients with TMJ ankylosis	CT scan, VR visualization, Brainlab	Standard CT guidance (Brainlab), additional VR guidance	OR time (sign. Reduction in patients with VR)	29
Franz et al.: A novel approach to skull-base and orbital osteotomies through virtual planning and navigation (51)	2019 The Laryngoscope	Pilot study	Skull base, orbit	Positioning of osteotomy-lines in orbital surgery with intraoperative navigation	3D slicer, CT scan	Optical navigation system StealthStation Treon cranial, (medtronic, Louisville, CO)	CT scan, deviation of >3 mm	15
Wei et al.: The safety and accuracy of surgical navigation Technology in the treatment of lesions involving the skull base (52)	2017 Journal of Craniofacial Surgery	Cohort study	Skull base (Oncology, TMJ)	Safety of navigated surgery at the skull base	CT, cbCT (0.2 mm)	Navigation system (Accu-navigation; 1.0 mm accuracy)	None	15
Dixon et al.: Augmented real-time navigation with critical structure proximity alerts for endoscopic skull base surgery (38)	2014 The Laryngoscope	Randomized Controlled Trial	Endoscopic surgery, pituitary surgery	Intraoperative usage of augmented reality	CT scan	Intraoperative usage of an alert in proximity to vital structures	Cadaver dissection trial	Fourteen cadaver specimen
Haq et al.: Single stage treatment of ankylosis of the temporomandibular joint using patient-specific Total joint replacement and VSP (50)	2014 British Journal of Oral and Maxillofacial Surgery	Cohort study	TMJ surgery	Successful treatment of TMJ ankyloses with virtually planned individual TMJ implants	CT scan, virtual planning (dolphin)	Cutting guide	Clinical evaluation	5
Haerle et al.: VSP in endoscopic skull base surgery (36)	2013 The Laryngoscope	Cohort study	Skull base surgery (endoscopic)	The value of VSP in skull base surgery	MRI (2 mm); Virtual (open source) vs. Conventional planning	Intraoperative navigation; StealthStation; med-tronic navigation, Louisville, CO	None	12
Tang et al.: Preoperative surgical planning for intracranial meningioma resection by virtual reality	2012 Chinese Medical Journal	Cohort	Skull base		CT, MRI, MRV Virtual reality			10

## Discussion

**Figure 1:**
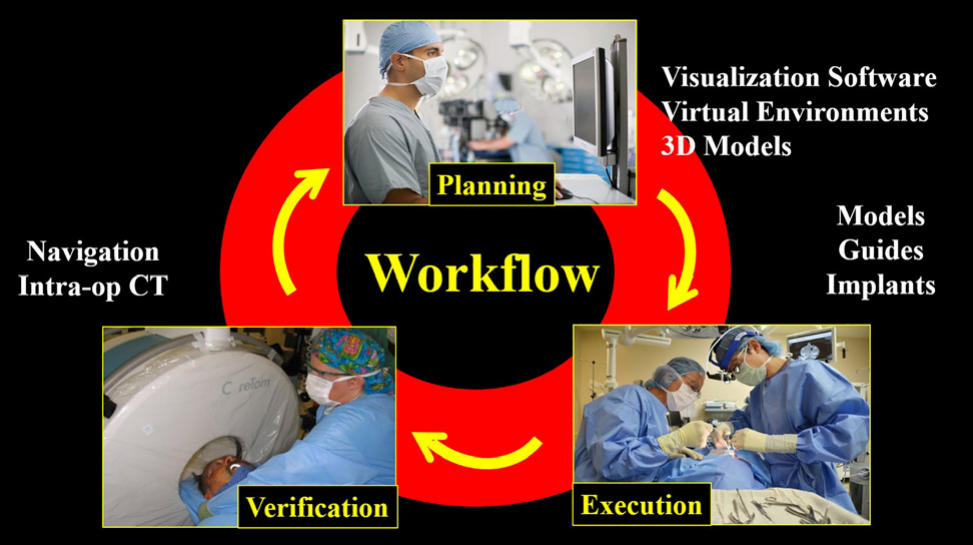
Computer aided surgical workflows generally involve three phases: Pre-surgical planning, surgical execution, and intra-operative verification.

Computer aided surgery can be divided into three phases: 1) **Virtual planning**, 2) **surgical execution**, and 3) **intraoperative verification** (Figure 1). Each of these will be discussed below.

### Virtual planning

CT is a critical CAS modality because it clearly defines bony margins and tumor infiltration. It is the primary modality for virtual surgical planning, 3D printing, and surgical navigation [25, 26]. CT imaging can be divided into two major categories: Fan beam CT which are often called “medical CT scanners,” and cone beam CT which are often called “in office” CT scanners [27]. Both modalities can be used for CAS applications [28]. Their advantages and disadvantages are discussed below (see *intraoperative *verification). Magnetic resonance imaging (MRI) has superior soft tissue resolution when compared to CT. However, poor bony resolution has made planning, navigation, and 3D printing more challenging. However, the pediatric skull base literature has recently described expansion of MRI for fabrication of 3D models and cutting guides, using the negative “black bone” sequences [29], [30], [31]. Refinement of this technique could significantly reduce radiation exposure while generating the necessary information for CAS applications.

**Figure 2:**
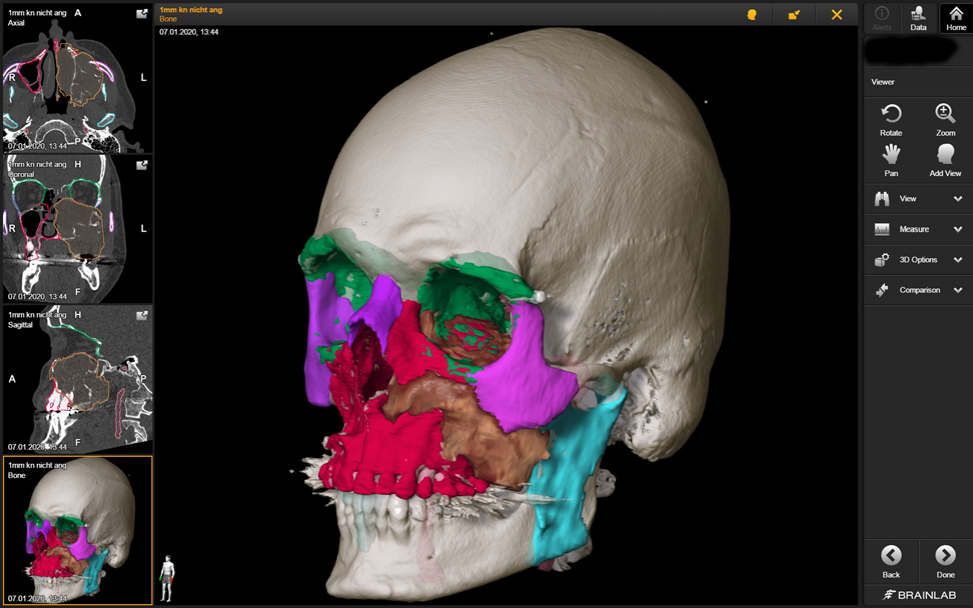
Segmentation of an invasive squamous cell carcinoma (brown) of the left maxillary sinus and adjacent structures (red – maxilla, green – orbit, pink – zygomatic bone, purple – nasal bone and contralateral zygomatic bone).

Virtual planning tools include 1) planning software, 2) virtual environments, and 3) 3D models. Each of these will be discussed below:

**Virtual Planning software:** Virtual planning software converts DICOM (“DICOM” – **D**igital **I**maging and **Co**mmunication in **M**edicine) data, the common language used by medical devices [30], to a propriety format that generates a 3D patient representation. Several examples include Mimics (Materialise – Leuven, Belgium), Elements (Brainlab – Berlin Germany), Invivo6 (Anatomage, Santa Clara, CA, USA), and Dolphin (Dolphin Imaging & Management Solutions – Chatsworth, USA). Alternatively, open source software is available, but surgeons need to consider the clinical implications when using “non-CE or FDA” approved software tools. Planning software provides a myriad of functions including soft tissue/bony segmentation (Figure 2), measurement tools (distances/angles/volumes), object mirroring, and fusion of data sets (overlap of two CT scans or MRI and CT). 

Planning may be performed using a *surgeon-based model* which requires the surgeon to purchase, learn, and independently utilize the software; or an *industry-based model* where surgeons and clinical engineers meet in an online setting, incurring only the time commitment to the surgeon. Cost recovery from industry in this second model varies and may include fee for service or incorporation of engineer fees into the cost of implant production. Once planning is complete, data can be exported in proprietary or non-proprietary formats for use in virtual environments (see *augmented or virtual reality* below), 3D printing, implant fabrication, as well as intraoperative navigation.

**Figure 3:**
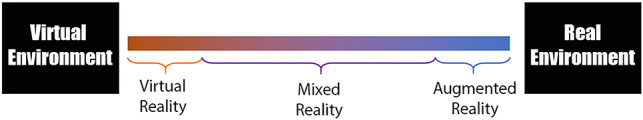
Reality spectrum: 1) virtual reality completely separates the user from the real world 2) augmented reality overlays information (data) onto the physical environment, 3) mixed reality allows the users to see their environment and places objects that can be manipulated within that environment.

**Figure 4:**
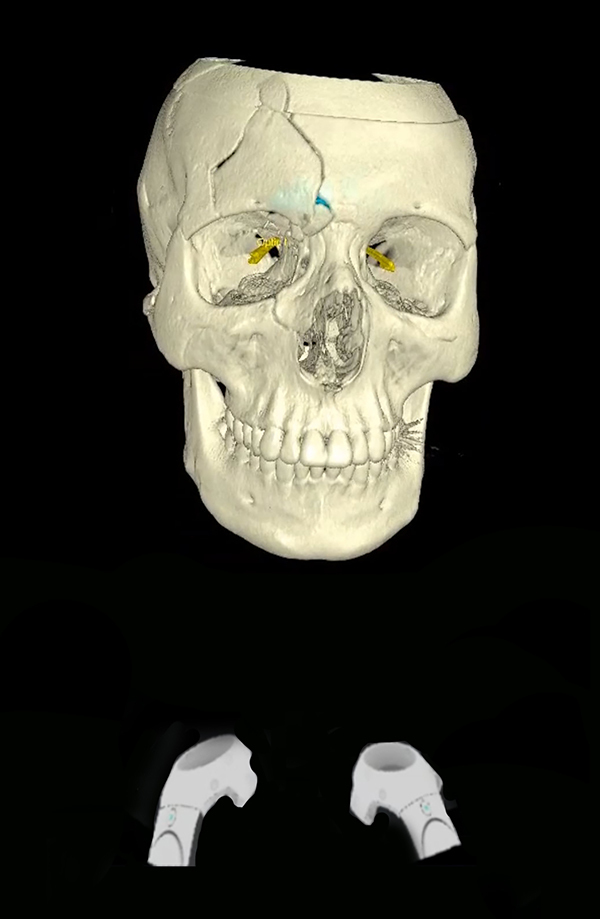
Virtual reality environments completely separate the user from their physical environment while allowing the user to manipulate virtual objects.

**Figure 5:**
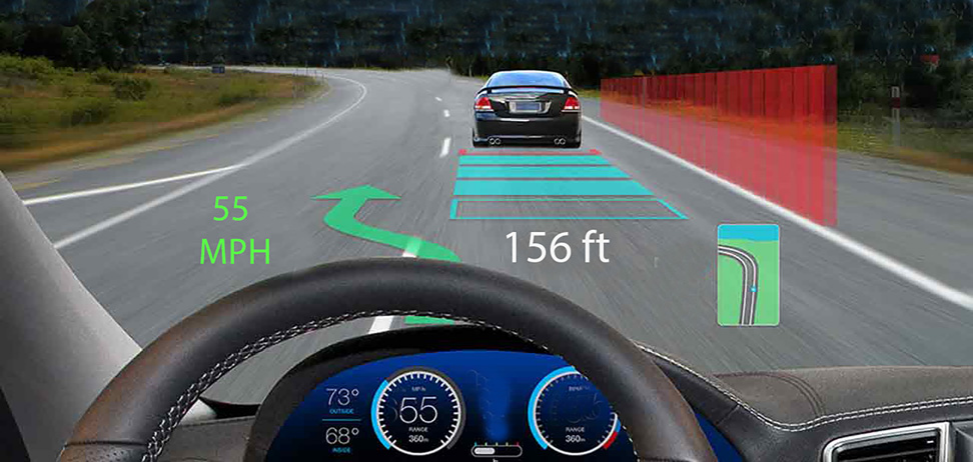
Augmented reality overlays information (often data) onto the environment as shown in this “heads up display”.

**Figure 6:**
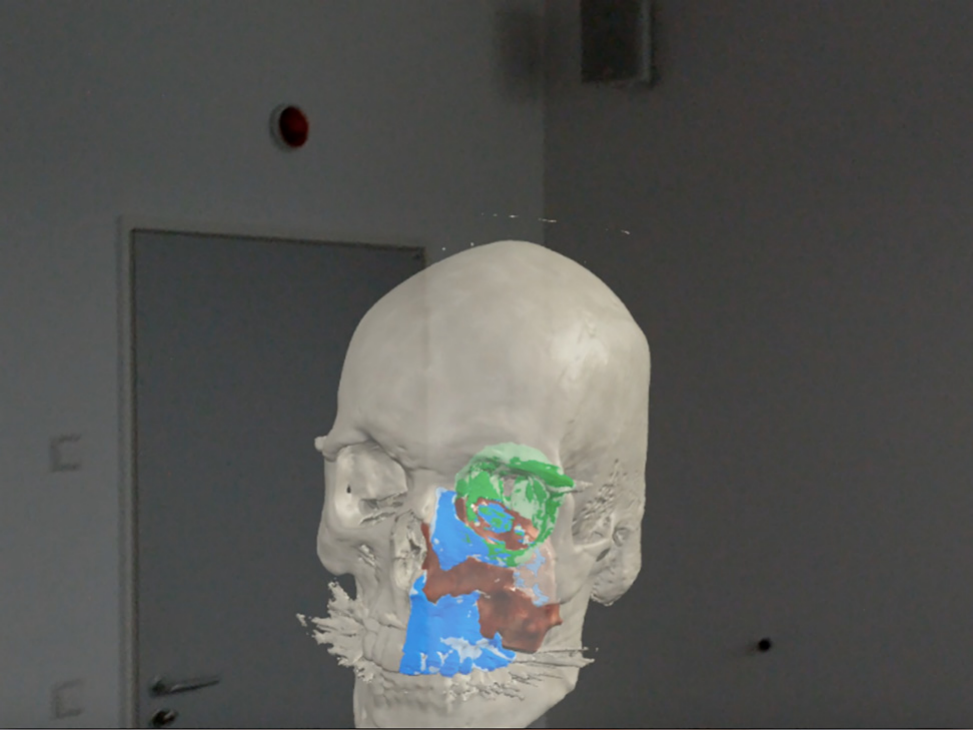
Mixed realith tools allow the user to visualize their environment, superimposing virtual objects into the environment that can be manipulated.

**Virtual Environments**: Virtual environments allow surgeons, trainees, as well as patients to visualize surgical anatomy in true 3-dimensional immersive space. These virtual environments can be described on a “reality spectrum” ranging between the “real environment” and a completely “virtual environment” (Figure 3). These tools can be divided into three basic types: ***virtual reality***, ***augmented reality***, and ***mixed reality***. *Virtual reality* tools completely separate the surgeon from his surrounding environment (Figure 4). This provides the user an extremely crisp and accurate view, however it makes interaction with other users more challenging and can lead to motion sickness [32, 33]. *Augmented reality* tools overlay information (generally data) onto the physical environment (Figure 5). *Mixed reality* tools allow the user to visualize their environment, superimposing virtual objects into the environment that can be manipulated (Figure 6). Objects appear more translucent (when compared to virtual reality), however users can easily interact with their environment, other users, and potentially the surgical field at some point in the future [34, 35]. Some examples include Magic Leap One Mixed Reality Viewer (Brainlab – Berlin Germany), Immersive Touch (Zimmer-Biomet – Warsaw, USA), and Precision VR (Surgical Theater – Cleveland, USA). Some tools include planning software which can be utilized by the surgeon (Magic Leap and Immersive Touch) while others (Precision VR) require an engineer to perform the surgical planning.

**3D Models**: 3D printing allows the surgeon to fabricate physical objects that can be used for surgical planning and pre-surgical simulation. This may include osteotomies, bony advancements/setbacks, resections, as well as trajectory planning and patient specific hardware bending.

#### Literature Review

**Bradley et al. 2019** – The authors evaluated the use of **virtual reality applications for TMJ** arthroplasty for congenital TMJ ankylosis. They compared two patients with traditional intraoperative CT based navigation to two patients treated using 3D VR guidance. They found no difference in complications or hospitalization times, but did report improved anatomic visualization and a statistically significant reduction in operative times (300 min for traditional CT navigation vs. 134 min with 3D VR guidance) [36].

**Haerle et al. 2013** – The authors describe the use of **VSP with 3D models in endoscopic anterior skull base surgery for sellar tumors**: Using ITK-SNAP 2.2 (University of Pennsylvania, Philadelphia, PA) to create 3D reconstructions of the sellar tumors and surrounding critical structures from MRI data, they found significantly reduced surgeon workload (as measured by the NASA-TLX questionnaire) during VSP and anatomic segmentation when planning skull base surgery compared to standard planning for both experts and novices [37].

### Surgical execution

Surgical execution tools allow the surgeon to perform surgical steps with improved accuracy and efficiency. Some examples include 3D Models, cutting/drill guides, surgical implants.

**Figure 7:**
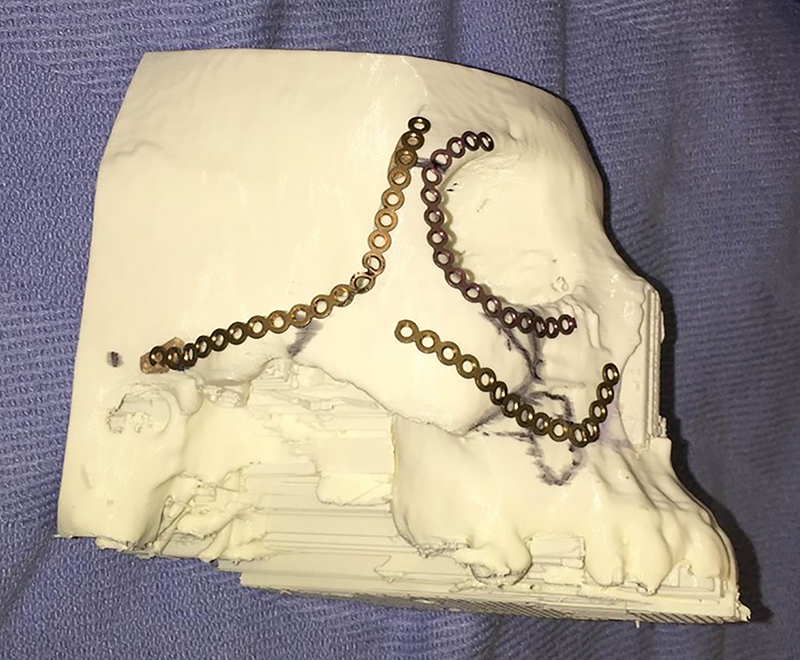
3D model used for template bending in the preoperative setting.

**3D Models**: Sterilization of 3D models for intraoperative use requires selection of materials that will withstand the autoclave process or selection of other low temperature sterilization processes such as Sterrad (Advanced Sterilization Products, Irvine, Ca, USA). Once sterilized, models can be used for intraoperative hardware bending (Figure 7), verification of anatomic landmarks, and resident education [18, 38].

**Figure 8:**
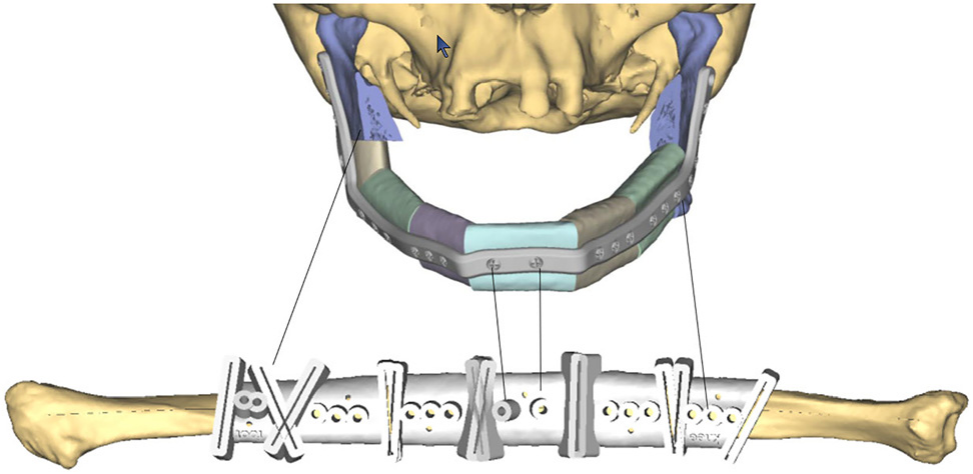
3D printed cutting guide for mandible reconstruction.

**Cutting/Drill Guides**: Intraoperative cutting and drill guides can be printed (or milled) for fibula free flap reconstruction (Figure 8), orthognathic surgery, and dental implants. Most cutting and drill guides are fabricated by industry with CE/FDA certification. They are very expensive, which has limited the total number of end users worldwide. Some institutions fabricate cutting and drill guides in house at a significantly reduced cost, however this incurs other potential costs including 3D printers/software, medicolegal risks, and surgeon time. Cutting and drill guides are particularly useful in planned (secondary) reconstructions, because there are clearly defined margins and the implant size is predictable. However, use in primary reconstruction is limited due to the unpredictablity of tumor resection margins. 

**Figure 9:**
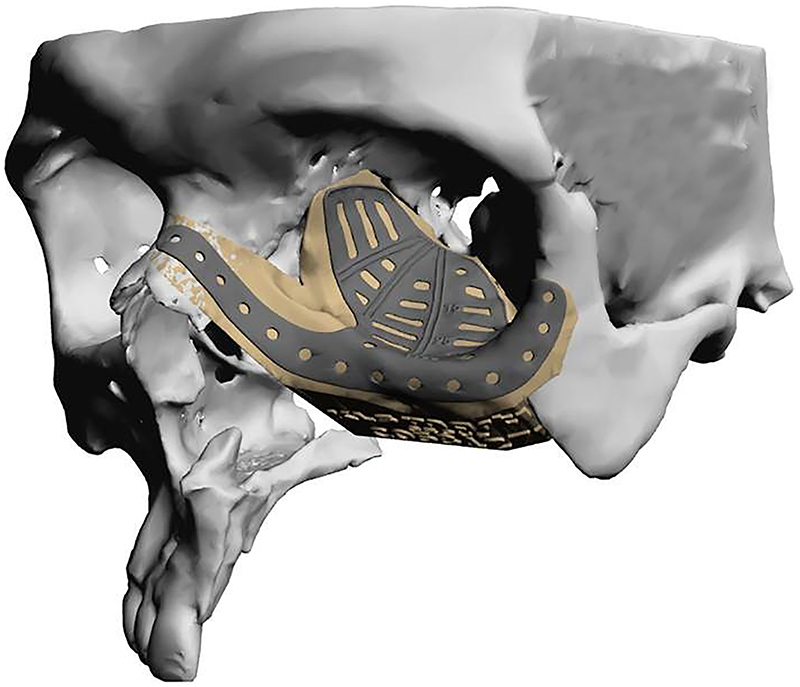
Commercially fabricated patient specific implant for orbito-midface reconstruction.

**Figure 10:**
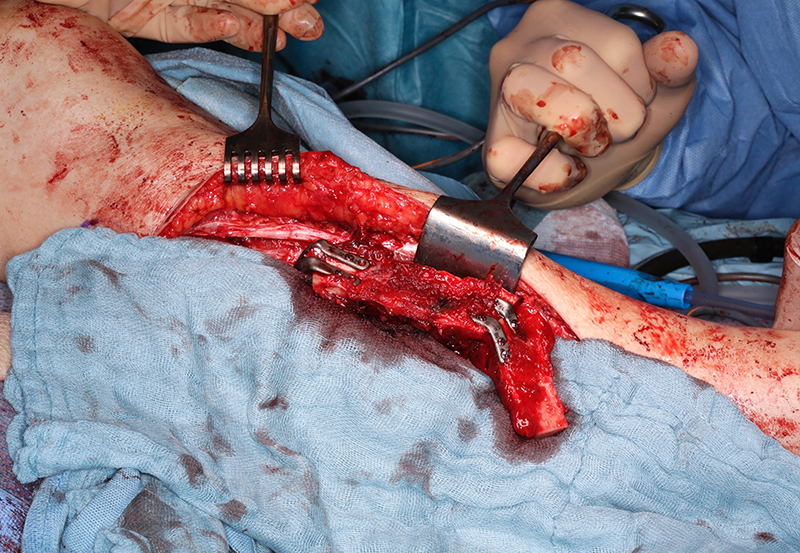
Patient specific implant used to shape fibula *in situ* for mandibular reconstruction.

**Surgical implants**: The use of 3D printing and milling allows for fabrication of patient specific implants (PSI). Some examples include orbital (Figure 9), TMJ, mandible (Figure 10), and cranial reconstruction. Due to strict manufacturing guidelines, the need for long term structural stability, and medicolegal risk, industrial partners generally fabricate these implants and few if any hospitals are entering into this arena. Potential drawbacks, especially in large implants (Figure 9) are imaging artifacts and implant exposure after radiation therapy. 

#### Literature Review

**Dixon et al. 2014** – In a cadaveric study, subjects (ENT Surgeons and Neurosurgeons) performed endoscopic transclival skull base approaches. The surgical limits were the carotid artery laterally, dura posteriorly, pituitary fossa superiorly, and the level of the petrous carotid inferiorly. Using VSP software and a prototype navigation system, vital structures (orbit, carotid, and dura) were highlighted preoperatively. Auditory feedback provided a warning when the surgeon was within 2–3 mm of the highlighted structures. The authors demonstrated that mental demand, effort, and frustration (as measured by the NASA-TLX score) were significantly reduced when using the VSP based system compared to standard navigation [39].

**Swendseid et al. 2020** – This retrospective cohort (n=23) study compared post oncologic scapular free flap midface reconstruction with and without the use of VSP. All 23 reconstructions used cutting guides. Fourteen procedures we performed without VSP and nine were performed with VSP. The authors demonstrated an improved reconstruction of subunits (98 vs. 78%), higher number of bone contact between bone segments (2.2 vs. 1.4 appositions), and higher percentage of segments placed in anatomic position (100 vs. 71%) in the VSP vs. non-VSP groups respectively [40].

### Intraoperative verification

After completion of many maxillofacial reconstructive procedures, visualization of the entire repair is challenging (ex. Orbital reconstruction, complex mandible reconstruction, zygomaticomaxillary reconstruction, etc.). Intraoperative navigation and CT can be efficacious to verify the accuracy of the reconstruction [41].

**Intra-operative navigation**: DICOM as well as proprietary data can be imported into surgical navigation systems. Once the data is imported, accurate “registration” of the patient is critical. The surgeon must provide the navigation system with precise landmarks on the patient’s skin, bone, oral appliance, or rigidly fixated hardware. Ideally, anatomic landmarks should be non-coplanar and non-collinear, collecting a sphere of points with the area of greatest interest at the center. This will allow the navigation system to accurately localize the patient in space, with the area of greatest precision being the area of interest (i.e. tumor, injury, anatomic deformity). The accuracy of most navigation systems ranges from 1 to 2 mm [42]. Registration modalities include electromechanical, electromagnetic, and optical [42]. Optical registration has several advantages for skull base reconstruction including: (1) it allows for free mobility around the patient without wires and (2) the instruments have no metallic distortion which can result in inaccuracies with electromagnetic systems [22, 43].

Once patient registration is complete, navigated instruments can be used to localize patient anatomy in difficult to visualize areas. It should be noted that all anatomic information provided by intraoperative navigation is “inferred.” In other words, it is only as precise as the imported data and accuracy of the registration process. Therefore, the information that is provided to the surgeon should be used to ***confirm*** anatomic information, not to ***guide*** surgical dissection.

Another advantage of navigation in oncologic surgery is the ability to place virtual “markers” within the 3D data set to identify the resection margins [22]. This information can then be used in combination with final pathology and radiation therapy to determine the optimal strategy for post-operative radiotherapy treatment.

***Intraoperative CT***: Unlike intraoperative navigation, CT incurs radiation exposure. Both fan beam and cone beam CT offer excellent intra-operative visualization, providing “real time” view of patient anatomy. However, there are some important differences to consider. Fan beam scanners provide excellent bone/soft tissue resolution and can be utilized with intravascular contrast material. However, they are much more expensive and incur a significantly higher radiation dose. Cone beam scanners have lower radiation dose, adequate bony detail, narrow slice thickness, and can be purchase for a much lower cost [27].

#### Literature Review

**Lee et al. 2012** – In this comparative cadaver study, the authors used an intraoperative C-arm to quantify surgical performance in anterior skull base surgery. They found significant improvement in point identification and line tracking tasks as well as improved accuracy of resection when using the intra-operative CBCT. Interestingly they demonstrated that in unguided cases (absence of intra-op CBCT), 35% of surgeons would have performed re-resection of residual tissue if they had not had access to Intraoperative CT [44].

**Novak et al. 2021** – This retrospective cohort study compared the accuracy of neuronavigation with preoperative imaging (CT & MRI), to neuronavigation using Intraoperative CT. They found significant improvement in accuracy when using Intraop CT as a basis for registration (mean error of accuracy 0 mm compared to 2.65 mm in pre-operative imaging data) [45]. They argue that intra-operative CT maintains excellent accuracy with lower radiation dose than traditional CT and no notable extension of surgical time.

## Conclusions

Computer aided surgery has three phases: 1) ***virtual planning***, 2) ***surgical execution***, and 3) ***intraoperative verification***. There is emerging data to show that these computer aided tools (presurgical planning software, augmented and virutal reality headsets, 3D printers, surgical navigation, and intraoperative imaging) improve surgical accuracy and clinical outcomes. Unfortunately, larger prospective studies are limited. As these applications continue to gain traction, the authors are confident that the surgical efficacy of these tools will continue to be validated.

## Supplementary Material

Supplementary Material
