# Supplementary material for: Computer assisted skull base surgery: a contemporary review
Source: Innov Surg Sci. 2022 Jun 30;8(3):149–57. doi: 10.1515/iss-2021-0020 (PMC10709692; doi:10.1515/iss-2021-0020)
Supplement: Supplementary file 1 — Supplementary Material [file j_iss-2021-0020_suppl.pdf]

Reviewer Assessment

A.K. Bartella et al.: Computer Assisted Skull Base Surgery: A Contemporary Review

Reviewers' Comments to Original Submission

Reviewer 1: anonymous

Date received: 10-Jan-2022
Reviewer recommendation: **Return to author for minor modifications**
Reviewer overall scoring: **Medium**

Assessment Form scores: 5 = High/Yes; 3 = Medium/Adequate; 1 = Low

Is the subject area appropriate for the journal		4			
Does the title clearly reflect the paper's content?		4			
Does the abstract clearly reflect the paper's content		4			
Do the keywords clearly reflect the paper's content?		4			
Does the introduction present the problem clearly?			3		
Are the results/ conclusions justified?			3		
How comprehensive and up-to-date is the subject matter presented?			3		
How adequate is the data presentation?		4			
Are units and terminology used correctly?		4			
Is the number of cases adequate?			3		
Are the experimental methods/ clinical studies adequate?			3		
Is the length appropriate in relation to the content?		4			
Does the reader get new insights from the article?			3		
Please rate the practical significance.			3		
Please rate the accuracy of methods.			3		
Please rate the statistical evaluation and quality control.			3		
Please rate the appropriateness of the figures and tables.		4			
Please rate the appropriateness of the references.		4			
Please evaluate the writing style and use of language.		4			
Please judge the overall scientific quality of the manuscript.			3		
Are the methods used worthy of reproduction in greater deal?	Yes				
Would you be willing to review a revision of this manuscript?	Yes				

Comments to author: We encourage you to include some more references and contents dealing with some other common procedures (e.g. central anterior/anterolateral craniofacial resection(as well as some discussion about image fusion. Distinction between resective and reconstructive surgical procedures / execution would be also desirable

Reviewer 2: Beat Hammer

Date received: 10-Jan-2022
Reviewer recommendation: **Accept in present form**
Reviewer overall scoring: **High**

Assessment Form scores: 5 = High/Yes; 3 = Medium/Adequate; 1 = Low

Is the subject area appropriate for the journal	5				
Does the title clearly reflect the paper's content?	5				
Does the abstract clearly reflect the paper's content		4			
Do the keywords clearly reflect the paper's content?	5				
Does the introduction present the problem clearly?	5				
Are the results/ conclusions justified?		4			
How comprehensive and up-to-date is the subject matter presented?			3		
How adequate is the data presentation?			3		
Are units and terminology used correctly?	5				
Is the number of cases adequate?					
Are the experimental methods/ clinical studies adequate?					
Is the length appropriate in relation to the content?			3		
Does the reader get new insights from the article?				2	
Please rate the practical significance.				2	
Please rate the accuracy of methods.		4			
Please rate the statistical evaluation and quality control.					
Please rate the appropriateness of the figures and tables.			3		
Please rate the appropriateness of the references.	5				
Please evaluate the writing style and use of language.	5				
Please judge the overall scientific quality of the manuscript.				2	
Are the methods used worthy of reproduction in greater deal?	Yes				
Would you be willing to review a revision of this manuscript?	No				

Comments to author: Good overview over the spectrum of computer assisted techniques used in skull base surgery Personally I try to avoid large implants as shown on page 23 . They tend to interfere with MRI follow up and in case of radiotherapy there is danger of exposure .

Recommendation by the Guest-Editor to the Original Submission

Revise with Minor Modifications

Authors' Response to Reviewer Comments

Date received: 02-Mar-2022

We would like to thank you for the thorough review of our manuscript. Your insightful comments have certainly helped in improving the quality of our paper, and we hope that our manuscript is now suitable for publication. According to your constructive advices, we have changed the paper.
In the point-to-point answers below we included the reviewers' comments in bold font, and our responses are subsequently stated in regular font. We also highlighted the changes we made in the manuscript.

Response to reviewer 1

We encourage you to include some more references and contents dealing with some other common procedures (e.g. central anterior/anterolateral craniofacial resection(as well as some discussion about image fusion. Distinction between resective and reconstructive surgical procedures / execution would be also desirable

We thank the reviewer for bringing up this important issue. As we understand it correctly, this article is part of a special issue addressing computer aided planning and computer assisted surgery. There will be also an article written from the perspective of an oral maxillofacial surgery, which will address central and anterolateral craniofacial resections. To prevent duplication of contents, we would suggest leaving this field to our colleagues. We added discussion about the differences between resection and reconstruction as suggested.

Response to reviewer 2

Good overview over the spectrum of computer assisted techniques used in skull base surgery. Personally I try to avoid large implants as shown on page 23 . They tend to interfere with MRI follow up and in case of radiotherapy there is danger of exposure.

We thank the reviewer for the constructive feedback. We agree with the concerns and added them to the discussion.

Comments by the Editor-in-Chief to Revised Submission – Final Decision

All reviewer comments were addressed by the authors. Reviewer raised only minor points for improvement on the original version. Therefore, I accept the revised manuscript for publication in our journal.
